# Immune-mediated gastritis in a patient with breast cancer due to therapy with immune checkpoint inhibitor pembrolizumab: a case report

**DOI:** 10.3389/fonc.2025.1624185

**Published:** 2025-10-10

**Authors:** Yalan Xu, Junyao Wang, Ning Chen

**Affiliations:** Department of Gastroenterology, Peking University People’s Hospital, Beijing, China

**Keywords:** gastritis, immune checkpoint inhibitors, pembrolizumab, glucocorticoids, case report

## Abstract

The use of immune checkpoint inhibitors (ICIs) in the treatment of malignant tumors is becoming more widespread, and the management of immune-related adverse events (irAEs) has become an important concern in oncological treatments. Here we present a 44-year-old female patient with triple-negative breast cancer (TNBC), who developed severe nausea and vomiting symptoms after pembrolizumab treatment. Gastroscopy showed extensive exfoliation, redness, and swelling of the gastric mucosa. Histology suggested complete destruction of the gastric mucosa and a large number of immune cells infiltration. After excluding other differential diagnoses, we considered the diagnosis of immune checkpoint inhibitor-associated gastritis. In responding to methylprednisolone treatment, her symptoms improved, and a repeated gastroscopy suggested repair in gastric mucosa, but demonstrated autoimmune gastritis-like manifestations. This case report describes a rare case of ICI-mediated gastritis. The importance of early endoscopy is emphasized and hormonal therapy remains the mainstay of treatment for this patient.

## Introduction

ICIs, including anti-cytotoxic T-lymphocyte-associated protein 4 (anti-CTLA-4) and anti-programmed cell death-1 (PD-1) receptors/programmed cell death-Ligand-1 (PD-L1) antibodies, are used to enhance anti-tumor immunity by blocking immune checkpoint proteins and are used in the treatment of many types of tumors, which has significantly prolonged advanced tumor patients’ survival and fundamentally changed the existing treatment paradigm for tumor patients (1, 2). Immunotherapy for breast cancer (BC) has also made significant progress in recent years with the approval of various ICIs, especially in early-stage and metastatic TNBC (3). However, in addition to the remarkable efficacy, immune-related adverse events (irAEs) have been observed in 30-60% of patients treated with ICIs, involving almost any organ of the body, including the skin, gastrointestinal (GI) tract, liver, thyroid, heart, etc. (4) Here, we present a rare case of ICI-associated gastritis with extensive exfoliation of gastric mucosa triggered by Pembrolizumab.

## Case presentation

The patient is a 44-year-old woman who was admitted to the hospital with “nausea and vomiting for more than 4 months”, accompanied by anorexia, upper abdominal pain, without obvious black stools or blood in the stool. She was diagnosed with TNBC 10 months ago, and was treated with totally 12 cycles of Pembrolizumab (the last dose was administered one month ago). She was diagnosed with *Helicobacter pylori* (Hp) infection but remained untreated for one year and had been diagnosed with psoriasis for many years. She denied any consumption of non-steroidal anti-inflammatory drugs (NSAIDs), alcohol, or narcotics, and she did not have a past history of diabetes mellitus or hypertension.

**Figure 1:**
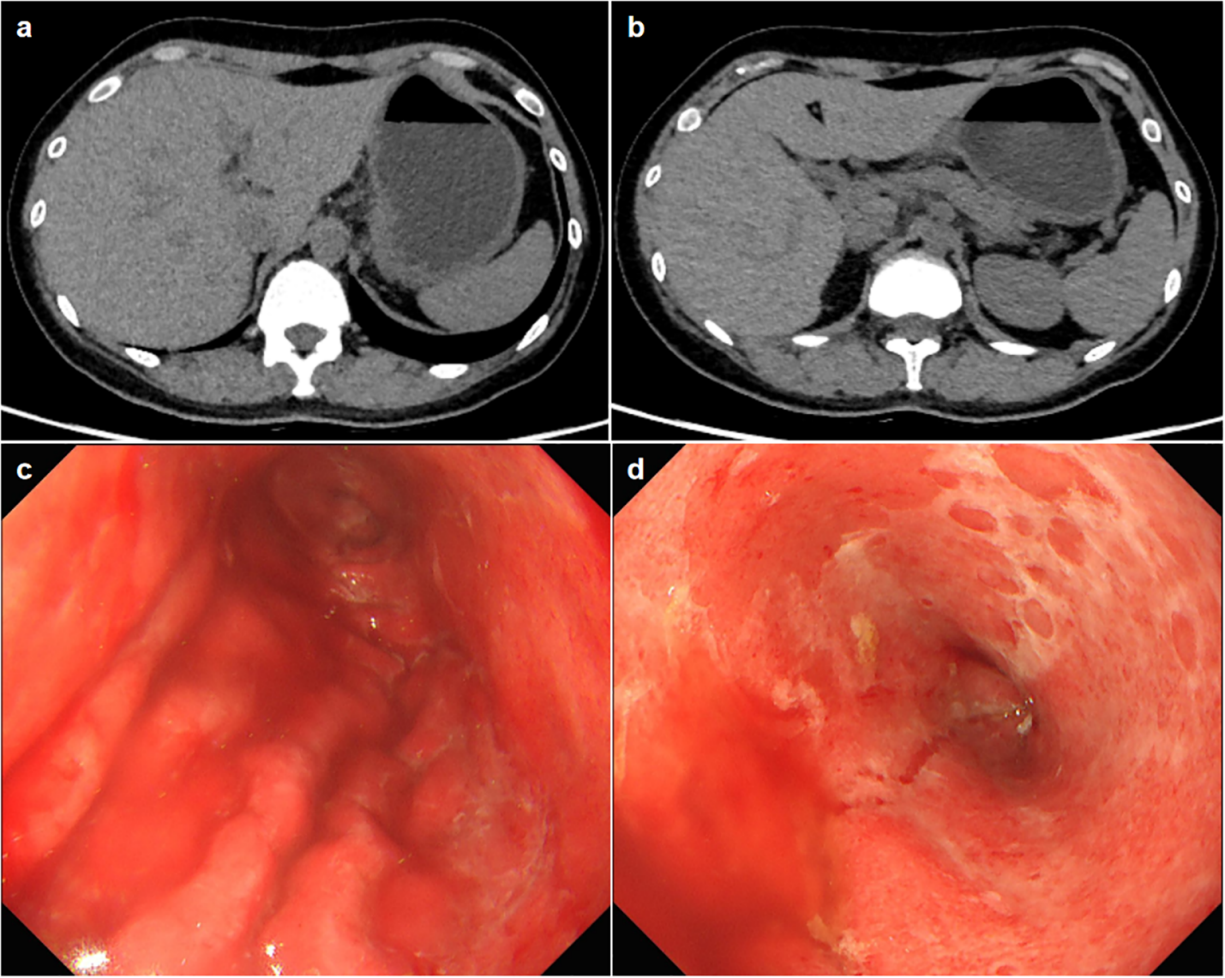
**(A, B)** Axial slice of computed tomography showing no thickening of the gastric wall or enlarged lymph nodes. **(C, D)** The esophagogastroduodenoscopy images show a widespread exfoliation of the gastric mucosa.

Physical examination showed vital signs were stable, only slight tenderness in the upper abdomen, but no rebound pain. Laboratory tests showed elevated alanine aminotransferase (161U/L, normal range 7–40 U/L), aspartate transaminase (222U/L, normal range 13-35U/L), Gamma-Glutamyl Transpeptidase (552U/L, normal range 7-45U/L), Alkaline phosphatase (546U/L, normal range 35-100U/L) and amylase in blood (249U/L, normal range 28-100U/L). Her blood sample was also tested positive for anti-Epstein-Barr(EB) virus chlamydial antigen(VCA) IgG and anti-EB virus nuclear antigen IgG, and an elevated concentration of EBV DNA (1.04×10^4^, normal value <4×10^2^ copies/ml) was detected. The complete blood count, C-reactive protein (CRP) and carcinoma embryonic antigen levels were normal. She tested negative for human immunodeficiency virus (HIV). Abdominal computed tomography did not demonstrate thickening of the gastric wall or enlarged lymph nodes in the abdominal cavity (**Figures 1A, B**). Magnetic resonance cholangiopancreatography showed a slight widening of the upper part of the common bile duct with a tortuous course, but no definite obstruction was found. The first look of her esophagogastroduodenoscopy in our hospital is as shown in **Figures 1C, D**. Endoscopic biopsy of the stomach suggested that the vast majority of glandular components disappeared and were replaced by inflammatory granulation tissue, with lymphocytes and eosinophilic granulocytes infiltration. Immunohistochemistry showed that CD3, CD4 and CD8 were positive. Rapid urease test of the stomach tissue was positive; The Epstein-Barr encoding region *in situ* hybridization suggested scattered positive. Cytomegalovirus was not found in the stomach tissue.

### Establishing the diagnosis

There are many factors that cause diffuse gastric mucosal injury, including drugs, alcohol, infection, malignancy, corrosive chemicals, and radiation therapy (5). Given that the patient is a cancer patient, having received multiple courses of chemotherapy and immunotherapy, she was vulnerable to infections that could involve the stomach. Given that HP urease test was positive and EBV DNA was detected in both her serum and gastric mucosa biopsy samples, a diagnosis of Hp gastritis or EBV-associated gastritis needed to be considered. However, the patient did not have any chicken-skinned appearance, crural enlargement, or turbid mucus in her stomach, which were regarded as typical HP gastritis manifestations (6). The patient’s presentation was not consistent with acute EBV infection, as evidenced by the lack of characteristic symptoms, normal inflammatory markers, and a negative EBV VCA-IgM serology. Consequently, the detected EBV is likely an incidental latent infection in the context of their comorbidities, not the cause of gastritis. The patient’s gastroscopic biopsy pathology, immunohistochemistry and special staining results did not reveal any other evidences of malignancy or systemic diseases for the time being; She had no history of corrosive chemical ingestion, gastric radiotherapy, NSAIDs and heavy alcohol consumption, no stressful events were experienced before the onset of the disease; thrombosis was absent on abdominal imaging; the autoantibody panel revealed an isolated positive anti-nuclear antibody (ANA) at a low titer of 1:40 and nti-parietal cell and anti-intrinsic factor antibodies were not detected, so that other factors causing diffuse gastric lesions were all excluded. At this time, we also noted that the patient had a clear history of ICIs treatment, and nausea and vomiting symptoms began to appear during the therapy, immunohistochemical findings in the affected mucosa indicate that T-cell-mediated immune killing contributes to mucosal tissue validation, injury, and necrosis, and laboratory tests also suggested injuries of other digestive systems, including elevated aminotransferase, cholestatic enzyme, lipase, and amylase levels. In combination with the previous literature reports (7–9), we considered that the patient might be more consistent with ICI-associated gastritis as a prominent manifestation of irAEs in the gastrointestinal tract. However, ICI-associated pancreatitis as well as ICI-associated liver injury cannot be excluded.

### Treatment outcome

For this patient, empirical treatment with methylprednisolone at a dose of 25 mg had been applied once daily intravenously for seven days, followed by an oral dose of 30 mg prednisone once every morning. Her symptoms significantly improved at the beginning but the condition worsened when she tried resumption of a semi-fluid diet. She experienced vomiting and abdominal pain again. It was worth noting that the patient’s peripheral blood was tested negative for EBV DNA spontaneously during the course of steroid therapy, which provided substantial confidence for the diagnosis of ICI-associated gastritis. Methylprednisolone was given at an increased dosage of 80 mg daily for four days and then followed by an oral dose of 50 mg prednisone. The discomforts were alleviated in 1 week. After discharge, careful tapering of prednisolone at 5 mg per week was recommended, and complete remission of GI symptoms was achieved.

**Figure 2:**
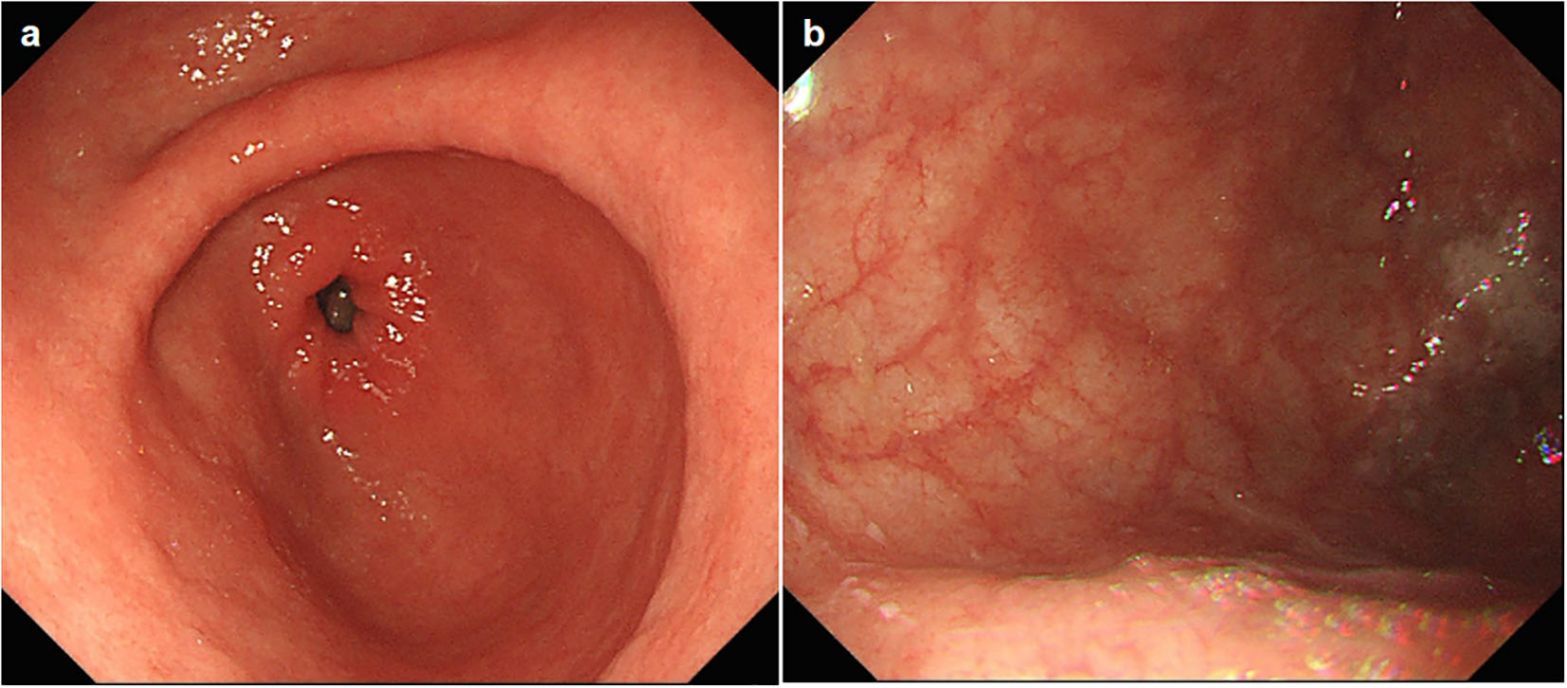
**(A, B)** The esophagogastroduodenoscopy images 3 months after discharge from hospital.

**Figure 3:**
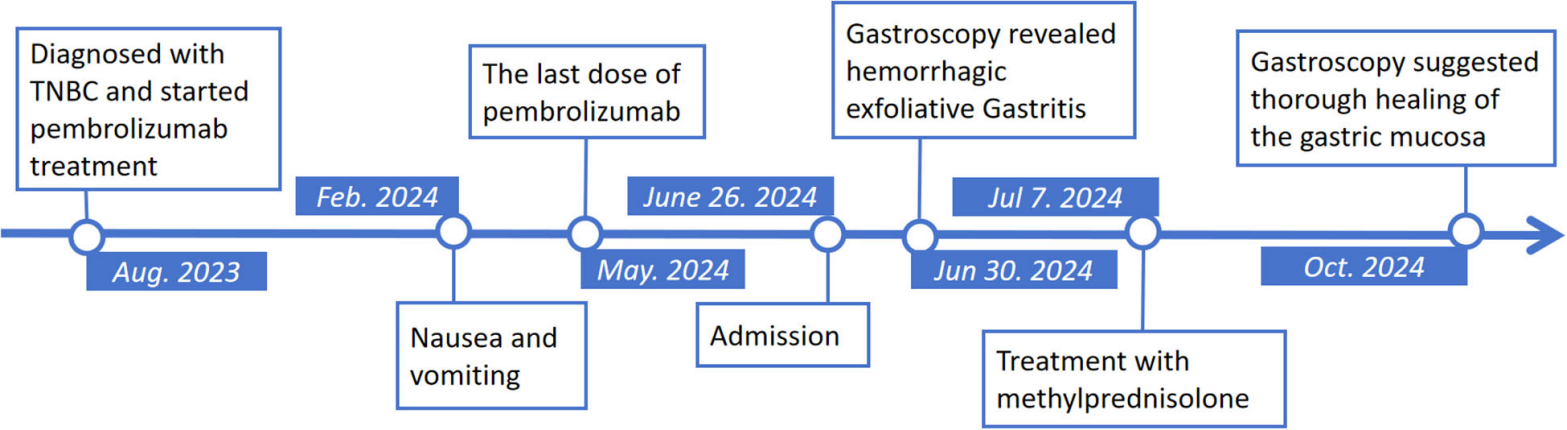
Timeline of patient treatment and examination.

A follow-up gastroscopy was performed about 3 months posterior to discharge, suggesting thorough healing of the gastric mucosa. Her stomach was otherwise normal except for large areas of atrophy in the gastric body and fundus (**Figures 2A, B**), demonstrating autoimmune gastritis. The patient permanently discontinued pembrolizumab (and all similar immunotherapy) and was switched to combination therapy with oral capecitabine and radiotherapy for breast cancer (**Figure 3**), and is ready for eradication of HP infection currently.

## Discussion

Among gastrointestinal injuries of irAEs, ICI-associated colitis is one of the most commonly reported one. Statistically, 5%-40% of ICI-treated patients develop ICI-associated colitis, and patients often present with diarrhea, abdominal pain, fever, blood in the stool and other symptoms (10). Comparatively, the incidence of ICI-associated gastritis is relatively low, with several retrospective studies based on large samples reporting a prevalence of approximately 0.35%-1.46% (11, 12), but it can lead to serious complications, such as gastric hemorrhage.

The mechanism by which ICIs trigger irAEs is unclear. It may include: imbalance of immune tolerance, cross-antigen presentation, production of multiple cytokines, off-target effects, and alterations in the microbiota (13). Both cellular and humoral immunity are involved in the development of irAEs.

The onset of ICI-associated gastritis ranges from 2 weeks to 156 weeks after initiation of immune therapy, with a median interval of 29.3 weeks. Symptoms are varied, with nausea, vomiting, and abdominal pain as common manifestations, sometimes accompanied by bloating and dyspepsia (14). Gastroscopy is a reliable means of diagnosis and manifestations such as erythema, edema, and ulceration of the gastric mucosa can be observed (15). Histopathological examination usually observes diffuse inflammation characterized by plasma cells and granulocyte infiltration with abundant eosinophils, and concurrent crypt abscesses in the epithelium and lamina propria. Serological findings and abdominal CT scan are not specific. Clinical management for ICI-associated gastritis is based on individualized assessment of clinical parameters. In asymptomatic patients, a wait-and-see approach may be appropriate. Immunotherapy is usually discontinued after the development of gastritis of grade 2 and above. In a few cases of isolated gastritis, symptoms improved with proton pump inhibitor therapy alone. However, in most cases, steroids are the first-line empirical medication. Immunosuppressive agents, such as TNF-α inhibitors (e.g., infliximab) or integrin blockers (e.g., vedolizumab) (16, 17), may be used steroid-resistant gastritis if there is no improvement within 2 to 3 days after intravenous steroid administration (18).

Diffuse gastric inflammatory lesions in patients undergoing ICI therapy continue to underscore the need to consider potential diagnosis of ICI-associated gastritis. As the clinical use of ICIs expands further, clinicians need to pay more attention to this category of adverse events and be sufficiently vigilant to avoid irreversible consequences. Gastroscopy is recommended as a first-line diagnostic modality, with prompt pharmacological intervention once diagnosed.

## Data Availability

The raw data supporting the conclusions of this article will be made available by the authors, without undue reservation.
